# Multivariate Statistical Analyses Demonstrate Unique Host Immune Responses to Single and Dual Lentiviral Infection

**DOI:** 10.1371/journal.pone.0007359

**Published:** 2009-10-06

**Authors:** Sunando Roy, Jennie Lavine, Francesca Chiaromonte, Julie Terwee, Sue VandeWoude, Ottar Bjornstad, Mary Poss

**Affiliations:** 1 Department of Biology, The Pennsylvania State University, University Park, Pennsylvania, United States of America; 2 Department of Statistics, The Pennsylvania State University, University Park, Pennsylvania, United States of America; 3 Department of Microbiology, Immunology, and Pathology, Colorado State University, Ft. Collins, Colorado, United States of America; 4 Fogarty International Center, National Institutes of Health, Bethesda, Maryland, United States of America; National Institute of Allergy and Infectious Diseases, United States of America

## Abstract

**Background:**

Feline immunodeficiency virus (FIV) and human immunodeficiency virus (HIV) are recently identified lentiviruses that cause progressive immune decline and ultimately death in infected cats and humans. It is of great interest to understand how to prevent immune system collapse caused by these lentiviruses. We recently described that disease caused by a virulent FIV strain in cats can be attenuated if animals are first infected with a feline immunodeficiency virus derived from a wild cougar. The detailed temporal tracking of cat immunological parameters in response to two viral infections resulted in high-dimensional datasets containing variables that exhibit strong co-variation. Initial analyses of these complex data using univariate statistical techniques did not account for interactions among immunological response variables and therefore potentially obscured significant effects between infection state and immunological parameters.

**Methodology and Principal Findings:**

Here, we apply a suite of multivariate statistical tools, including Principal Component Analysis, MANOVA and Linear Discriminant Analysis, to temporal immunological data resulting from FIV superinfection in domestic cats. We investigated the co-variation among immunological responses, the differences in immune parameters among four groups of five cats each (uninfected, single and dual infected animals), and the “immune profiles” that discriminate among them over the first four weeks following superinfection. Dual infected cats mount an immune response by 24 days post superinfection that is characterized by elevated levels of CD8 and CD25 cells and increased expression of IL4 and IFNγ, and FAS. This profile discriminates dual infected cats from cats infected with FIV alone, which show high IL-10 and lower numbers of CD8 and CD25 cells.

**Conclusions:**

Multivariate statistical analyses demonstrate both the dynamic nature of the immune response to FIV single and dual infection and the development of a unique immunological profile in dual infected cats, which are protected from immune decline.

## Introduction

Infections with both human and feline immunodeficiency viruses cause progressive deterioration of immune responses and are characterized by decline of CD4 cells. Although circulating CD4 T cell count is an excellent indicator of disease progression, the mechanisms involved with control of HIV and FIV and maintenance of immunological competency are still under active investigation. In part, this is because host and virus factors that affect disease outcome are complex and change over time, which makes it difficult to identify specific parameters responsible for immunological health in infected individuals. However, there are several lines of evidence that suggest protection from the pathogenic consequences of HIV-1, SIV or FIV is achievable. First, there are intriguing reports that the asymptomatic period of HIV-1 infected persons may be prolonged in individuals concurrently infected with distantly related HIV-2 [1]. Second, the most effective lentivirus vaccine trial to date are with modified live viruses in the rhesus macaque model [2]. Last, primary infection of cats with FIV derived from naturally infected wild cougars also confers protection against disease caused by virulent domestic cat FIV [3], [4]. Significantly, in the SIV/ macaque experimental model and the natural FIV cat system, protection can occur without clear indicators of, or correlation with, specific anti-viral responses [3], [5], [6]. Although preventing infection is a principal goal, understanding how a primary infection with an attenuated or genetically distant virus can ameliorate the pathological consequences of a more virulent virus would have significant impact on therapeutic strategies.

FIV infection of cats provides an important animal model to address this significant question because cats are the only hosts that develop an immunodeficiency syndrome from a natural lentivirus infection that can be used for experimental studies. Previous studies have established that a primary infection with cougar-derived strain PLV-1695 (PLV hereafter) protects cats from CD4 decline caused by subsequent infection with a virulent feline immunodeficiency virus (FIVfca) strain (FIVC36; FIVC hereafter) [3]. Both experimental and natural infections of cats with FIVC lead to a fatal immunodeficiency syndrome [7] similar to that observed in humans infected with HIV-1 [8], [9]. PLV-infected cats develop low level viremia after an initial peak of virus replication [10], [11] but there are no clinical manifestations of PLV infection in domestic cats or in the natural cougar host [12]–[14]. Though primary PLV infection did not elicit antibody or cellular adaptive responses that provided protection to FIVC infection, of importance, CD4 cell depletion was abrogated in dual, but not single, FIVC infection [3], [11]. Initial analyses of a complex data set comprised of relevant immune soluble and cellular factors indicated that IFNγ differed significantly among cats with single and dual infections at some time points. However, no other clear association of immune parameters with viral infection status was detectable using univariate statistical methods [3].

We undertook the present study because, despite the broad recognition of the dynamic and inherently multivariate nature of the immune response, immunological studies often resort to simple univariate statistical techniques (such as t-tests or analysis of variance) applied separately to each response parameter in the experimental data [15]. Such analysis may obscure, or fail to reveal, important features of the data. A rich set of multivariate analysis methods exists in the statistical literature and they are frequently applied in other fields (e.g. ecology or environmental sciences) to investigate complex systems (e.g. comprising individuals or molecules with overlapping functions, amplifying downstream effects of small perturbations, etc.). Indeed, greater insight into the immune response is reached where appropriate statistical analyses are employed [16]–[19].

It is of great therapeutic importance to understand the immune environment that is associated with disease attenuation in chronic lentivirus infections. Thus, we conducted a multivariate analysis on the measured immune parameters collected during the first four weeks following inoculation with virulent FIVC into naïve and PLV infected animals. The clinical parameters of this study, outlined briefly here, are reported in detail in TerWee et al. [3]. Our aim in the present analysis was to ascertain whether the primary immune response to FIVC was affected by the presence of PLV using a suite of multivariate statistical methods that are suitable to explore data for typical experimental infection studies; those with small sample size, missing values, and co-variation among measured parameters. We pursued this with a three-fold strategy: (*i*) we quantified and characterized the co-variation among responses, (*ii*) we investigated significant effects of infection and co-infection on these responses, and, (*iii*) we identified combinations of responses that discriminate among infection groups. By drawing composite immunological profiles of the different infection groups, our analyses demonstrate that these profiles do indeed distinguish groups at several time points during the first weeks of infection with virulent FIVC. Importantly, we identify an immunological response in dual infected cats that is unique from those mounted to either single infections with exotic PLV or virulent FIVC that suggests underlying mechanisms for disease abrogation.

## Materials and Methods

### Study Design and data Pre-processing

The experimental design is described in detail by Terwee et al. [3]. Two groups of 10 cats were sham inoculated or inoculated with PLV at day 0. At day 28, five cats in each group were inoculated intravenously with FIVC. Thus, we investigated four infection groups, each containing five animals: two single infected groups (one with FIVC and one with PLV), one group infected with both PLV and FIVC (dual infected) and one uninfected group. Using the approaches described in [3], eleven immune response parameters were measured on the 20 cats; namely, CD25, neutrophils, CD8, CD4, TNFα, Lymphocytes, IL12, IL10, IL4, IFNγ and FAS (see Table 1 for a list and descriptions). This was performed repeatedly at four time points post-FIVC infection; namely, days 31, 37, 52 and 59, referenced to the start of the experiment; these are days 3, 9, 24 and 31 post-FIVC challenge. Values for the immune parameters are also available at day 45, but we excluded this day because 5 of the 20 cats were missing cytokine measures.

**Table 1:** List of immunological response parameters used in the analysis.

Symbol	Description
Th-1 and Th-2 cytokines:	Soluble factors modulating innate and adaptive immune response
IL-4	B-cell growth factor, ‘Th2’cytokine
IL-10	B-cell survival and proliferation, ‘Th2’. Generally antagonistic to TNFα
IL-12	Stimulates production of IFN-γ and TNFα, ‘Th1’
TNFα	Stimulates systemic inflammation, regulates apoptosis, neutrophil chemoattractant
IFNγ	Proinflammatory cytokine, stimulates IL-12 and TNFα, antagonistic to IL-4, ‘Th1’
FAS	‘Death receptor’, induces apoptosis
Circulating immunocytes:	Peripheral markers of immune homeostasis
Lymph	T and B Lymphocytes, NK cells and monocytes
CD4	Cell surface marker for T helper cells (lymph subset)
CD8	Cell surface marker for cytotoxic T cells (lymph subset)
CD25	Cell surface marker for activated T cells (both CD4 and CD8) and T regulatory cells
Neutr	Neutrophils; granular leukocytes, phagocytic. (innate immune system)

This dataset was not complete for each value at each time point due to limitations of sample availability. From a total of 55 values for each infection group at each time point; day 31 had 14 (of 55) missing values for the uninfected group and 7 (of 55) missing from the FIVC group; day 37 had no missing values; day 52 had 1 (of 55) missing value for the FIVC group and 1 (of 55) for the PLV group; day 59 had 6 (of 55) missing values for the PLV group. We used *Nonlinear Iterative Partial Least Squares* (NIPALS; see http://biomserv.univ-lyon1.fr/~dray/files/softwares/nipals.R [20] to impute missing values – the *nipalsPCA* function from the *pcaMethods* package of the R language [21] produced a full “reconstructed” data set.

Since our immune responses are expressed in different measurement units and present very different spreads, we also applied a data *Normalization* at the outset; for each day and each response, we subtracted the mean and divided by the standard deviation. This was done for all 20 cats, and thus represents an overall re-centering and re-scaling of the data, which does not affect relative positions and spreads of the four infection groups.

### Statistical Analyses

To address challenges set out in the Introduction, we employed a few broadly used multivariate techniques, which we summarize here and detail below:

- The levels of many proinflammatory cytokines and immunocytes in our data do co-vary, i.e. have a systematic tendency to move upward or downward of their averages together (positive co-variation) or in opposite directions (negative co-variation). The nature of this co-variation is informative, and can be quantified and characterized through *Pearson's correlation coefficients*. In addition to studying the pair-wise correlations, we also performed a spectral decomposition of the Pearson's coefficients matrix – which is equivalent to applying *Principal Component Analysis* (PCA [22], [23]) to the normalized data.
- To investigate significant effects of single and dual infection on the responses, we used *Multivariate Analysis of Variance* (MANOVA; [22], [23]) with tests based on Pillai's trace. Since the responses have a marked co-variation structure, these provide enhanced power relative to univariate tests assessing differences in infection group means separately for each response [24].
- To identify combinations of responses that discriminate among infection groups, we used *Linear Discriminant Analysis* (LDA; [22], [23]. Instead of analyzing the responses as a function of the infection groups, this analyzes the groups, as labeled, as a function of the responses – which here take the role of a vector of discriminating features. LDA produces linear combinations of immune response parameters, interpretable as “immune profiles”, which best distinguish among infection status.

### Principal Component Analysis

**Figure 1:**
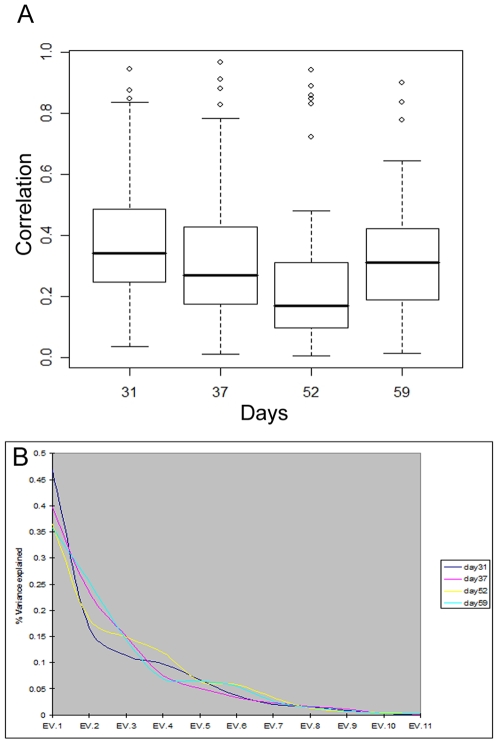
(A) Box-plots of (absolute) pair-wise correlations and (B) Scree plots from PCA. (A) The horizontal axis indicates the four days of analysis, and the vertical axis shows the distribution of absolute pair-wise correlations among the 11 immunological response parameters. Open circles indicate parameter pairs with particularly strong correlations (“outliers” in the box-plots). Note that on day 52, the majority of parameters have lower correlations overall, but there are several that are highly correlated (anti-correlated). (B) The ordered PCA components are shown on the horizontal axis (EV stands for eigenvector). The vertical axis shows the share of overall variability explained by each component, in different colors for each of the four days of analysis.

PCA is a method to extract directions, i.e. linear combinations of variables, which are most relevant to the variability of a multi-dimensional data cloud. PCA is based on the spectral decomposition of the variance-covariance matrix of the data and produces a set of orthogonal eigenvectors, each identifying a direction, associated with eigenvalues listed in decreasing order. The eigenvalues represent variances of the data along the directions described by the corresponding eigenvectors/linear combinations. Thus, the first eigenvector (linear combination) identifies the direction of largest variability, the second the one of next largest variability, subject to the constraint of being orthogonal to the first, etc (see Figure 1). In addition to the *pair-wise* measures of linear association provided by the entries of the variance-covariance matrix, the relative size of its leading (largest) eigenvalues capture the degree of linear interdependence among the variables *as a group*. For the analysis of immunological responses, interdependencies can be crucial since the immune system has many feedback loops that cause cell and cytokine levels to be associated.

PCA is used as a dimension reduction and data visualization technique. If a few leading eigenvalues strongly dominate all others, the data is well captured (in terms of its variability structure) by a projection on the space spanned by the corresponding eigenvectors. Consequently, in many applications, PCA can be used to produce 2 or 3-dimensional plots that provide a satisfactory visualization of high-dimensional data. PCA for each of four days in our study was carried out on the normalized data (i.e. in terms of the correlation matrix) using the *princomp* function from the *stats* package of the R language [21].

### Multivariate Analysis of Variance

MANOVA is the multivariate analogue of an Analysis of Variance (ANOVA) model, and allows a simultaneous comparison of the means of several responses across “treatment groups”, taking into account their co-variation – as represented by the error variance-covariance matrix in the MANOVA model. Tests for mean differences between two groups are usually based on Hotelling's T^2^ statistic. When the problem comprises more than two groups, as is the case in our study, several statistics can be used, including Pillai's trace, Wilk's λ, Lawley-Hotelling's trace, Roy's Greatest root, etc. The resulting tests are generally in accord with each other; we focused on Pillai's trace, since it provides multivariate tests that are robust to differences among the error variance-covariance matrices within groups, as well as to departures from the assumption of multivariate normality [24], [25].

When assessing group differences in a multivariate setting, MANOVA can avoid misleading conclusions associated with the use of univariate t- or F-tests for individual responses. Intuitively, the reasons for this are that (*a*) univariate tests do not account for co-variation among responses, and (*b*) in addition to better power due to accounting for co-variation, multivariate tests allow a better handle of false positives (whose joint probability increases when performing multiple univariate tests) [21], [26].

Two-way MANOVA models (PLV infection  =  Yes, No, crossed with FIVC infection  =  Yes, No, including overall level, main effects of each infection and interaction effect) were fitted for each of the four days on the normalized data using the *manova* function from the *stats* package of the R language [21]. In particular, for each day and each effect, we computed Pillai's trace-based p-values. For comparison with the MANOVA, we also fitted two-way ANOVA models separately for every immunological response using the *aov* function in R. In particular, for each day, response and effect, we computed F-based p-values. This produced 11 ANOVA p-values (one for every response) for each MANOVA p-value, so the former were adjusted with a simple Bonferroni correction (i.e. multiplied by 11).

### Linear Discriminant Analysis

Like PCA, LDA is a method to extract directions, i.e. linear combinations of variables. However, in LDA the directions are selected based on their ability to separate labeled groups in a multi-dimensional data cloud [27]. The spectral decomposition of a matrix is employed also here; while PCA uses the variance-covariance matrix of the data, LDA uses the *between groups* variance-covariance matrix normalized against the *within groups* variance-covariance matrix. Again, the technique produces a set of orthogonal eigenvectors (each identifying a direction) associated with eigenvalues listed in decreasing order; the first eigenvector (linear combination) identifies the direction of maximal group separation, the second the one of next maximal separation, subject to the constraint of being orthogonal to the first, etc., where group separation is defined benchmarking between group against within group variation.

Whereas with MANOVA we model our immunological parameters as a function of the infection status, with LDA we analyze infection status as a function of the immunological parameters, which here represent *discriminating features*. LDA, therefore, allows us to extract “immune profiles” (combinations of the response parameters) that provide maximal separation among infection status. LDA, like PCA, can be used for dimension reduction and data visualization. If a few leading eigenvalues strongly dominate all others, the data is well captured (in terms of group separation) by a projection on the space spanned by the corresponding eigenvectors.

**Figure 2:**
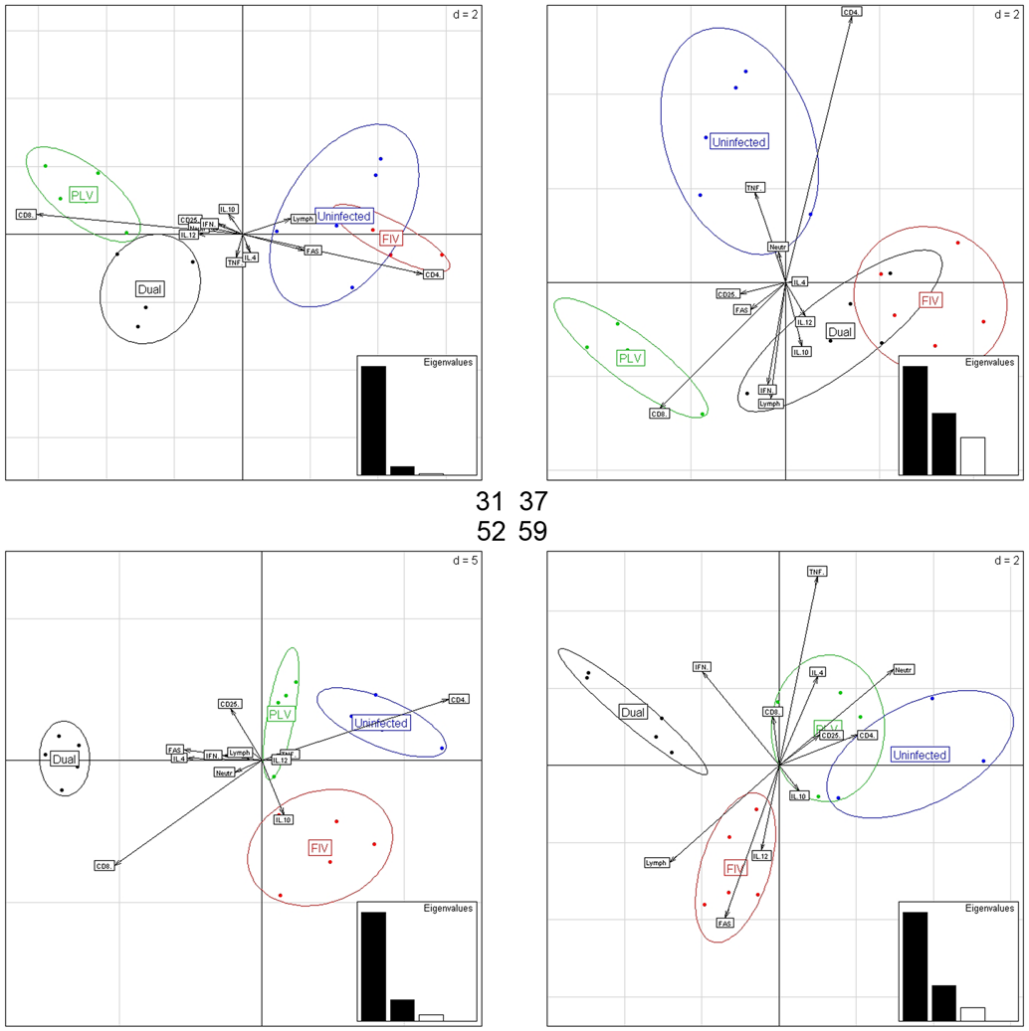
Bi-plots from LDA, for days 31 (upper left), 37 (upper right), 52 (lower left) and 59 (lower right). The horizontal and vertical axes on each panel represent first and second LDA direction, respectively. The arrows depict loadings of the 11 responses; their orientation and length indicate the role of each immunological parameter in relation to the LDA directions and the different infection groups, on each of the analysis days. The insets in each panel show first, second and third LDA eigenvalue (discriminatory importance of the corresponding LDA directions).

LDA for each of the four days was carried out on the normalized data using the *lda* function from the *MASS* package of the R language [21]. Since our focus was on the association between immune response parameters and infection status, we produced projective graphics called *bi-plots* obtained through LDA. These LDA bi-plots (see Figure 2) comprise: (i) Projections of the data points, marked by groups, on the plane spanned by the first and second LDA directions. (ii) Elliptical contours capturing each group's variability in the plane of the bi-plot. (iii) Loadings for the 11 immune response parameters, i.e. coefficients with which the responses enter LDA directions (which help us interpret the “immune profiles” we extracted); each response is represented as an arrow in the bi-plot, with orientation expressing signs, and horizontal and vertical sizes proportional to the loadings of the feature relative to the first and second LDA directions, respectively. (iv) Eigenvalues associated with LDA directions, i.e. the discriminatory “contribution” of these directions; each eigenvalue is represented as a bar in an inset graph – black bars are eigenvalues for first and second directions, which span the plane of the bi-plot, and the white bar is the eigenvalue for the third direction, which is not rendered in the bi-plot (since there are four groups in our study, all subsequent eigenvalues are 0 by construction). In addition to standard plotting commands, the functions *s.class* and *s.arrow* from the *ade4* package of the R language [21] were used in implementing the bi-plots.

## Results

### Immune response parameters co-vary

We expect substantial interdependencies for the immunological parameters in our study (Table 1). For instance, some responses will positively co-vary because they are subsets (CD4 and CD8 cells are both lymphocytes), or if there is a primary B cell proliferative response – Th2 – (IL4 and IL10), or a proinflammatory response – Th1 – (IL12, TNFα, IFNγ, FAS). Moreover, cytokines classified as ‘Th1’ often will negatively co-vary with those classified as ‘Th2’, but in some cases cytokines such as IFNγ and IL10 will positively co-vary [28] due to co-expression associated with regulation of the immune response. Thus, individual cytokine levels can be less informative on the nature of the immunological response at any time point than the suite of cells and cytokines.

The 11 immune parameters we considered do indeed present strong co-variation at each of the four times. We summarized pair-wise linear associations computing the four correlation matrices (Pearson's correlation coefficients), and performed PCA on each. Figure 1, upper panel, shows box-plots of the absolute values of the correlations at days 31, 37, 52, and 59 – the mean (median) correlations at these days are 0.39 (0.34), 0.33 (0.27), 0.27 (0.17) and 0.33 (0.31), respectively. The absolute correlations are well removed from 0 with numerous large values; the majority of these correlations are statistically significant (see Tables 2 and 3). Figure 1, lower panel, shows the shares of variability explained by each principal component at days 31, 37, 52, and 59. The leading eigenvalues dominate strongly in each day, with percent of explained variability for first (second) components equal to 47% (17%), 40% (24%), 36% (19%) and 36% (26%), respectively. This indicates a large degree of linear interdependence among the variables as a group, at all four times. The bulk of the (absolute) correlation values and the dominance of the leading eigenvalues decrease from day 31 to 52. In particular, days 52 and 59 present the lowest level of co-variation, while having a few very strong pair-wise associations.

**Table 2:** Pearson's coefficients of the ten strongest (absolute) pair-wise correlations between immunological responses for each day of analysis.

Day 31	Correlation Pair	Day 37	Correlation Pair	Day 52	Correlation Pair	Day 59	Correlation Pair
**0.603**	**FAS,** a **IFNγ**	0.574	IL4, IL12	b,c−0.352	Lymph, TNFα	−0.469	IFNγ, CD25
0.605	IFNγ, CD8B	0.638	IL10, IL12	c −0.355	IL10, Lymph	−0.486	IL10, Lymph
0.702	IFNγ, TNFα	0.714	IL12, TNFα	0.480	*IL10, TNFα*	0.567	IFNγ, IL4
0.808	CD8, CD25	0.718	*IL10, TNFα*	**0.722**	**FAS, IFNγ**	−0.583	FAS, Neutr
0.820	CD4, CD25	0.754	CD4, CD25	0.829	Lymph, CD25	0.583	FAS, TNFα
0.831	Lymph, CD25	0.782	CD8B, CD25	0.848	CD8B, CD25	−0.645	IFNγ, Neutr
0.836	*IL10* a, *TNFα*	0.828	Lymph, CD25	0.858	CD4, CD25	0.777	CD4, CD8
0.847	Lymph, CD4	0.881	Lymph, CD8	0.888	Lymph, CD8	0.778	Lymph, CD8
0.873	Lymph, CD8	0.910	Lymph, CD4	0.941	Lymph, CD4	**0.836**	**FAS, IFNγ**
0.943	CD4, CD8	0.966	CD4, CD8	0.941	CD4, CD8	0.898	Lymph, CD4

a Pair-wise correlations that increase (bold) or decrease (italics) are highlighted.

b The sign indicates whether a pair co-varies positively or negatively.

c All coefficients are statistically significant (p-values substantially smaller than 0.05) using a test for association/correlation between paired sample, except for the two negative correlations of Lymph with TNFα and IL10 at day 52, (TNFα p-value  = 0.13, IL10 p-value  = 0.12).

**Table 3:** Spearman's coefficients of the ten strongest (absolute) pair-wise correlations between immunological responses for each day of analysis.

Day 31	Correlation Pair	Day37	Correlation Pair	Day52	Correlation Pair	Day59	Correlation Pair
0.67	CD4, CD25	0.51	IL12, TNFα	b−0.50	IL10, CD4	0.51	CD4, Neutr
0.67	IL10, TNFα	0.53	IL10, IL12	0.54	Lymph, CD25	−0.51	FAS, CD25
0.67	IFNγ, CD8	**0.65**	**FAS, IFNγ**	0.54	Lymph, CD8	0.52	Lymph, Neutr
**0.68**	**FAS, IFNγ** a	0.71	IL10, TNFα	0.55	IL4, IL12	0.52	Lymph, CD8
0.68	Lymph, CD4	0.86	Lymph, CD8	−0.61	IL10, Lymph	−0.55	IFNγ, CD25
0.71	Lymph, CD25	0.88	CD8, CD25	**0.61**	**FAS, IFNγ**	−0.60	FAS, Neutr
0.78	CD8, CD25	0.89	Lymph, CD4	0.64	CD4, CD25	0.61	CD4, CD8
0.79	Lymph, CD8	0.90	CD4, CD25	0.68	CD8, CD25	−0.66	IFNγ, Neutr
0.80	IFNγ, TNFα	0.92	Lymph, CD25	0.72	Lymph, CD4	0.84	Lymph, CD4
0.89	CD4, CD8	0.97	CD4, CD8	0.79	CD4, CD8	**0.88**	**FAS, IFNγ**

a Pair-wise correlations that increase are bolded.

b The sign indicates whether a pair co-varies positively or negatively.

c All coefficients are statistically significant (p-values substantially smaller than 0.05) using a test for association/correlation between paired sample.

We further explored the ten strongest pair-wise correlations at each time to identify immunological parameters with the most marked associations (Table 2; this represents approximately the top 20% of the correlations in each day – the reported values are all statistically significant, except for two, as noted in Table 2). There is a strong positive correlation between total lymphocyte counts and both CD4 and CD8 counts at all time points. CD4 and CD8 levels also show strong positive correlation at all time points. We note that CD4 and CD8 are cell surface receptors expressed on T lymphocytes, and that CD25 can be expressed on both CD4 and CD8 cells if they are activated. Thus, strong positive correlations of these cellular markers are to be expected if there is a general expansion of T lymphocyte subsets. In fact, the strongest correlations occur among cellular parameters for days 31, 37, and 52. The correlation between FAS and IFNγ is consistent and increases with time from the FIVC infection, peaking at day 59 (bold in Table 2).

Since our data comprises only 20 observations (cats), we also considered *Spearman's correlation coefficients*, which are a robust version of Pearson's, computed on the ranks instead of the measured levels. Similar to the Pearson's, the mean (median) Spearman's correlations at days 31, 37, 52 and 59 are 0.41 (0.39), 0.29 (0.21), 0.27 (0.24) and 0.32 (0.32), respectively. Exploring the ten strongest Spearman's pair-wise correlations at each time (Table 3) also produces results consistent with Pearson's (Table 2).

It is important to note that our co-variation analyses concern the complete dataset from all 20 cats and do not capture or exploit specific relationships of immunological parameters with the infection groups; therefore the strong interdependencies detected through both pair-wise correlations and PCA confirm the need for multivariate techniques in investigating relationships between these immune parameters and infection status.

### Mean immune responses differ significantly across infection status

To investigate significant effects of infection and co-infection on the immune response parameters, and more specifically to assess differences between group means, we fit a MANOVA model for each of the four days considered. The model expresses our vector of 11 responses as a function of the PLV and FIVC infection status in a 2-way scheme comprising the four infection groups. In this manner, the effect of the two PLV-infected groups (PLV infection only and dual infection) can be compared with non-PLV infected groups (FIVC infection only, and uninfected) and *vice versa*. Tests based on Pillai's trace statistics can therefore be performed for the simple effect of PLV, the simple effect of FIVC and the “interaction” effect. Table 4 contains estimated effects and corresponding p-values.

**Table 4:** Estimated effects (E) and Pillai's trace p-values (p) for a two-way MANOVA scheme comprising simple and interaction effects of PLV and FIVC, for each day of analysis.

	Day 31	Day 37	Day 52	Day 59
PLV	**p = 0.006** a	P = 0.16	**p = 0.01**	p = 0.18
	**E = 0.95**	E = 0.81	**E = 0.93**	E = 0.80
FIVC	p = 0.52	P = 0.07	**p = 0.02**	**p = 0.04** b
	E = 0.65	E = 0.86	**E = 0.91**	**E = 0.89**
PLV × FIVC	p = 0.96	P = 0.29	p = 0.18	p = 0.56
	E = 0.36	E = 0.75	E = 0.80	E = 0.63

a Significant results are in bold.

b This value is not significant if the MANOVA is run without imputing the values (p = 0.06).

The simple effect of FIVC is significant at day 52 and 59 but not at days 31 and 37. Conversely, the simple effect of PLV has the highest significance at day 31, is significant at day 52 and is not significant at days 37 and 59. Our MANOVA testing does not detect a significant interaction effect at any of the times considered. However, the interaction p-value is smallest at day 52. We also conducted the MANOVA on the data without missing value imputation. This decreased the sample size by different amounts for each day (See Methods). The p-value for the effect of FIVC at day 59 increased slightly (from 0.04 to p = 0.06), and the p-value for the interaction effect of PLV and FIVC at day 52 decreased from 0.18 to 0.06 (the omitted imputed values here involved one animal each from the FIVC and PLV group). In all, the data highlights day 52 (day 24 post FIVC infection) as the one in which multivariate responses are most affected by infection status.

For comparison with the above multivariate results, we also fit 11 univariate 2-way ANOVA models for each of the four days considered. Here the model expresses an individual response as a function of the PLV and FIVC infection factors in the same 2-way scheme, and F-tests were performed for the simple effect of PLV, the simple effect of FIVC and the interaction effect. For each effect, and each day, we therefore obtained 11 p-values – one for every response; Table 5 lists response names, estimated effects and p-values for the cases that remained significant after Bonferroni correction.

**Table 5:** Estimated effects (E) and Bonferroni corrected F-based p-values (p) for two-way ANOVA schemes applied to each immunological parameter separately, for each day of analysis.

	Day 31	Day 37	Day 52	Day59
PLV	a IL-4:	IFNγ:	IFNγ:	IFNγ:
	p = 0.026; E = 0.45	p = 0.001; E = 0.62	p = 0.05; E = 0.40	p = 0.02; E = 0.48
	IFNγ:			
	p = 0.001; E = 0.62			
FIVC	None	CD25:	IFNγ:	CD25:
		p = 0.0002; E = 0.70	p = 0.02; E = 0.47	p = 0.05; E = 0.40
		CD8:	FAS:	Neutr:
		p = 0.00005; E = 0.74	p = 0.02; E = 0.46	p = 0.0002; E = 0.70
		CD4:		IFNγ:
		p = 0.0005; E = 0.66		p = 0.001; E = 0.63
		Lymph:		FAS:
		p = 0.0005; E = 0.66		p = 0.03; E = 0.48
PLV × FIVC	None	None	None	None

Only significant effects are reported, with the name of the corresponding parameters.

a This value is not significant if ANOVA is run without imputing the values (p = 0.13).

Similar to the MANOVA results, interaction effects are not detected as significant for any of the responses, at any of the times considered. However, a number of simple effects of PLV and FIVC status do survive the correction for multiple testing at each time. In particular, the effect of PLV is always significant in terms of the IFNγ levels although significance declines at days 52 and 59 in this group. In contrast, the effect of FIVC is significant in terms of both IFNγ and FAS at days 52 and 59 (recall these responses present a very strong positive correlation at these times; Table 1). In addition, cell phenotype parameters indicative of activated lymphocytes (CD25, CD8, CD4, lymphocyte) at day 37, and neutrophil counts at day 59, show a significant effect of FIVC. Thus, the ANOVA tests detect the strongest effect of FIVC infection at days 37 and 59 and indicate IFNγ as a “driver” of the immune response to PLV infection at all time points. The role of the immunophenotype is confined to day 37 (day 9 post-FIVC infection) as a simple effect of FIVC infection. Also here, we conducted the ANOVAs without missing value imputation, obtaining similar results, with the exception PLV did not have a significant effect for IL4 when omitting the missing values. In conclusion, both ANOVA and MANOVA tests suggest that there are differences in immune responses of groups with primary FIVC and PLV infection. However, some critical differences exist in the outcome of univariate and multivariate analysis approaches, which will be interpreted further in the Discussion.

### Specific immune profiles, which evolve over time, discriminate among infection status

To identify combinations of responses that discriminate among the infection groups (“immune profiles”), we performed LDA for the four groups on the 11 responses, for each of the four days considered. LDA output for days 31, 37, 52, and 59 is summarized in the four bi-plots in Figure 2. On day 31 (3 days post-FIVC infection; upper left panel) there is essentially one discriminating signal in the data, with a strongly dominating eigenvalue associated with the first LDA axis, which separates PLV infected (single and dual) and non-PLV infected (uninfected and FIVC-infected) cats. The feature loadings indicate that this corresponds to a “trade-off” between CD8 and CD4 cell numbers, with other immunological parameters playing secondary roles. It is also noteworthy that PLV infection induces an elevation in neutrophil count, CD25 cells, IFNγ, and IL12 compared to uninfected or recently FIVC-infected cats at this time point. Note that while some separation between FIVC-infected and uninfected cats, and single and dual PLV infected cats, exists along the second LDA axis, the eigenvalue associated with this axis is much smaller than the first.

By day 37 (9 days post-FIVC infection; Fig. 2, upper right panel), the picture has become more complex. The data contain at least two discriminating signals (see eigenvalues in the in-set), and the four groups separate along both the first and the second LDA axes. There is complete separation of single PLV- and FIVC-infected cats along the first LDA axis due primarily to higher expression of CD8, CD25 and FAS in PLV infected cats. FIVC dual and single infected cats show a substantial overlap in the projection on the first two LDA directions. Notably, the second LDA direction separates uninfected from all infected cats (PLV single, FIVC single, and dual); uninfected cats have higher CD4, TNFα and neutrophil levels, and lower CD8, lymphocyte, IFNγ and IL10 than any of the infected groups.

The discriminating signals captured by the first and second LDA directions provide maximal resolution of the four groups at day 52 (24 days post-FIVC infection; Figure 2, lower left). In particular, at day 52 we observe a clear “immunological profile” for dual infected cats, as they cluster far away from uninfected and both groups of single infected cats. Based on the loadings, this profile is characterized by higher CD8 and CD25 counts, and elevated levels of IL4, FAS, and IFNγ than that of the other groups. FIVC single infections separate from uninfected cats and single PLV infections due to higher levels of IL10 and lower CD4 counts. PLV infections separate from uninfected cats principally on the basis of lower CD4 counts.

The four treatment groups are also distinguishable at day 59 (31 days post FIVC-infection; Figure 2, lower right). Dual infected cats separate from the other three groups due to higher IFNγ levels and CD8 and lymphocyte counts. Single FIVC infected and dual infected cats separate along the second axis. Dual infected cats have elevated levels of TNFα, IL4, and IFNγ and CD8 cells and neutrophils compared to single FIVC infected animals. Single FIVC infections are characterized by higher IL12 and FAS levels and lymphocyte counts than dual infections. Uninfected and PLV single infected cats overlap in the projection on the first two LDA axes; both have higher levels of CD4 and CD25 cells and neutrophils compared to cats with single and dual FIVC infection.

The ellipses in the bi-plots of Figure 2, which represent the variance-covariance structure of the projected data points comprising the four groups, show marked differences across groups at each time and across times for each group. In particular, in these projective representations the uninfected group often appears more variable than the infected groups. However, these ellipses are not indicative of overall variability in the 11-dimensional response space. When considering all 11 dimensions, the uninfected group is indeed the tightest, with a total variance (sum of the 11 variances) that is fairly constant over time, whereas the infected groups show higher and changing total variance (Table 6). This indicates that infection status may affect not just the location, but also the variability structure of the 11 immunological measures considered here.

**Table 6:** Total variances within infection status groups on different days.

	Day 31	Day 37	Day 52	Day 59
Dual	14.26	12.36	9.29	10.43
PLV	12.54	7.2	15.95	9.73
FIVC	4.18	8.16	6.81	6.45
Uninfected	5.55	3.76	5.82	4.56
All cats	11 a	11	11	11

a The total variance for all cats (last row) is 11 on each day because of normalization.

## Discussion

In this study, we used multivariate statistical techniques to evaluate the immunological responses of cats infected with an apathogenic FIVC (FIVpco strain PLV-1695 derived from a cougar), virulent FIVC, or both viruses. FIVC infection on its own causes immunosuppression and death [7]. However, cats infected first with PLV and then with FIVC are protected from these consequences [3]. Immunological parameters measured on the cats consisted of lymphocyte expression levels for five cytokines, total number of circulating lymphocytes and neutrophils, three functional markers displayed on T lymphocytes, and a cell surface molecule regulating cell survival. Detecting differences among infection groups, as well as the discriminatory roles of various immune parameters, is complicated by interdependencies among these parameters, their diverse kinetics, and difficulties in gauging variability given the small sample size at our disposal. Employing well established and straightforward multivariate statistical techniques such as PCA, MANOVA and LDA, despite the limited number of observations, we found clear evidence that the response variables measured in our study do distinguish the four experimental groups.

Understanding the immunological response in cats infected with both PLV and FIVC is of particular interest in this study because dual infected cats are protected from FIVC-induced disease. Our data show that simultaneous infection with the two viruses elicits an immune response that is substantially different from that mounted to uninfected or single infection with either the apathogenic PLV or the virulent FIVC. FIVC infection is initiated at day 28 post-PLV infection. Notably, the immune profile of dual infected cats resembles that of PLV, not FIVC, 3 days post-FIVC infection (day 31). As FIVC infection proceeds to day 37, the immunological profile of all but one of the dual infected cats starts to resemble, or overlap with, that of FIVC infected cats. This highlights both the variation in cat responses at this date (see Table 6) and the dynamic nature of the immune response to a mixed infection. By day 52 (24 days after FIVC infection) the immunological response in dual infected cats is clearly distinct from all groups and is dominated by an elevated level of CD8, CD25, and FAS expressing cells, and increased numbers of lymphocytes and neutrophils. This phenotype may reflect expansion of an important effector T cell subset in these cats. IL4 and IFNγ are the only cytokines that contribute to the unique immune profile of dual infected cats at day 52. The dynamic nature of host response to dual virus infection is apparent in comparing the profiles of days 52 and day 59. For the first time, IFNγ is clearly a driver in dual infected cats at day 59 although the overall profile is not typically pro-inflammatory. In addition, increased lymphocyte counts with lower levels of CD4 and average levels of CD8 cells compared to uninfected and PLV infected cats suggest that B cells or NK cells may be increased in dual (and to a greater extent in single FIVC) infected cats at this time.

How does the immunological profile of cats infected with FIVC in the presence of a preexisting PLV infection differ from cats infected with FIVC alone? In general, single FIVC infection elicits an overall increase in IL10, IL4, and IL12, but lower expression of CD25, and CD8 throughout the first 31 days of infection (day 31–59 of the experiment) compared to other groups. Prior to day 59, FAS levels are lower in single FIVC infected cats but are increased at day 59 of the experiment. Neutrophil and CD4 levels also are lower at day 59. In contrast, the immune response in dual infected cats is an activated cellular profile maturing over the 31 days of dual infection. It is noteworthy that the differences in the immune response to FIVC in dual and single infected cats likely arise during the first week of infection because after that point, the profiles of PLV and uninfected cats are very similar. In addition, while FIVC infection induces CD4 decline and dual infected cats are protected from CD4 loss [3], CD4 is not associated with the dual infection group in the first three weeks of infection. In contrast, elevated levels of CD8 cells are consistently associated with PLV and dual infected cats over this time frame.

We show that the immunological parameters considered in our study present a marked co-variation structure; an indication that multivariate statistical methods are needed to derive a clear association between infection state and immune response. MANOVA tests evaluate whether there are significant effects of the treatment groups on the vector of response parameters as a whole. Using a two-way scheme, we demonstrate that there is a significant effect of PLV infection on immune parameters on day 31. This is also demonstrated by the LDA results from day 31, which show a clear separation of PLV and dual infected groups from FIVC and uninfected cats. Cell count responses, which have high positive correlations at all times, and in particular CD4 and CD8 counts, emerge as key differentiators between PLV and non-PLV cats at day 31. Importantly, neither CD4 nor CD8 carry significant effects in their individual ANOVAs for day 31 (Table 5). Also the difference CD8-CD4, which captures the dominant trade-off shown by the LDA loadings in Figure 2 (upper left) has no significant effect in an individual ANOVA for this day (data not shown). Yet, in combination with the other responses, this trade-off has a crucial role in group separation.

Our MANOVA results also indicated that there were significant effects of both FIVC and PLV infection on day 52 and a comparatively weaker role of the FIVC infection at day 59, both of which are consistent with the LDA findings. By contrast, univariate analysis (ANOVA) identifies the strongest effect of FIVC infection at days 37 and 59. Further, the immune parameters that emerge as significant in the univariate analysis (ANOVA) are not necessarily those that discriminate the four groups as determined by our multivariate approaches. For example, at day 52, when the effects of the dual infection are most pronounced based on MANOVA and LDA, ANOVA results show a marginally significant difference for IFNγ between PLV and non-PLV infections, and significant differences for IFNγ and FAS between FIVC and non-FIVC infections, suggesting that IFNγ is a strong driver of group discrimination. However, LDA reveals a more complex interplay among the immunological parameters at day 52. Higher levels of CD8 cell and lymphocyte and neutrophil counts, enhanced expression of FAS, IL4, and IFNγ, and lower levels of IL10, TNFα and CD4 cell counts are all associated with the separation of dual infected cats from all other groups. Most important, we are able to determine that the immunological response to single FIVC infection was relatively stable - dominated by IL-10 and low CD25 and CD8 - in contrast to the dynamic and pro-inflammatory profile seen in the dual infected cats over the first month following FIVC infection.

Although we qualitatively compared output of multivariate analyses at different time points, because of the small sample size of this study, we limited ourselves to relatively simple techniques, and did not venture into the more complex territory of multivariate methods and models that would allow us to include time as an endogenous factor in the study. We also did not attempt to account for heterogeneity we observed in the variability structure of the immune responses within each infection group. Methods and models to deal explicitly with dynamics and heteroschedasticity do exist [29]–[32], and we are planning to investigate their use on more complete data sets in the future.

## References

[1] Travers K, Mboup S, Marlink R, Gueye-Nidaye A, Siby T (1995). Natural protection against HIV-1 infection provided by HIV-2.. Science.

[2] Koff WC, Johnson PR, Watkins DI, Burton DR, Lifson JD (2006). HIV vaccine design: insights from live attenuated SIV vaccines.. Nat Immunol.

[3] Terwee JA, Carlson JK, Sprague WS, Sondgeroth KS, Shropshire SB (2008). Prevention of immunodeficiency virus induced CD4+ T-cell depletion by prior infection with a non-pathogenic virus.. Virology.

[4] VandeWoude S, Hageman CA, O'Brien SJ, Hoover EA (2002). Nonpathogenic lion and puma lentiviruses impart resistance to superinfection by virulent feline immunodeficiency virus.. J Acquir Immune Defic Syndr.

[5] Friedrich TC, Watkins DI (2008). Wanted: correlates of vaccine-induced protection against simian immunodeficiency virus.. Curr Opin HIV AIDS.

[6] Lifson JD, Rossio JL, Piatak M, Parks T, Li L (2001). Role of CD8(+) lymphocytes in control of simian immunodeficiency virus infection and resistance to rechallenge after transient early antiretroviral treatment.. J Virol.

[7] de Rozieres S, Mathiason CK, Rolston MR, Chatterji U, Hoover EA (2004). Characterization of a highly pathogenic molecular clone of feline immunodeficiency virus clade C. J Virol.

[8] Burkhard MJ, Dean GA (2003). Transmission and immunopathogenesis of FIV in cats as a model for HIV.. Curr HIV Res.

[9] VandeWoude S, Apetrei C (2006). Going wild: lessons from naturally occurring T-lymphotropic lentiviruses.. Clin Microbiol Rev.

[10] Poss M, Ross HA, Painter SL, Holley DC, Terwee JA (2006). Feline lentivirus evolution in cross-species infection reveals extensive G-to-A mutation and selection on key residues in the viral polymerase.. J Virol.

[11] Terwee JA, Yactor JK, Sondgeroth KS, Vandewoude S (2005). Puma lentivirus is controlled in domestic cats after mucosal exposure in the absence of conventional indicators of immunity.. J Virol.

[12] Biek R, Rodrigo AG, Holley D, Drummond A, Anderson CR (2003). Epidemiology, genetic diversity, and evolution of endemic feline immunodeficiency virus in a population of wild cougars.. J Virol.

[13] Biek R, Ruth TK, Murphy KM, Anderson CR, Johnson M (2006). Factors associated with microparasite seroprevalence and exposure risk in Rocky Mountain cougars.. J Wildlife Disease.

[14] Biek R, Ruth TK, Murphy KM, Anderson CR, Poss M (2006). Examining effects of persistent retrovirus infection on fitness and pathogen susceptibility in a natural feline host.. Canadian Journal of Zoology.

[15] Genser B, Cooper P, Yazdanbakhsh M, Barreto M, Rodrigues L (2007). A guide to modern statistical analysis of immunological data.. BMC Immunology.

[16] Keil D, Luebke RW, Ensley M, Gerard PD, Pruett SB (1999). Evaluation of multivariate statistical methods for analysis and modeling of immunotoxicology data.. Toxicol Sci.

[17] Collette A, Bagot S, Ferrandiz ME, Cazenave PA, Six A (2004). A profound alteration of blood TCRB repertoire allows prediction of cerebral malaria.. J Immunol.

[18] Lionel A, de Seze J, Didier L, Sandrine F-N, Sylvain D (2005). Evolution of self-reactive IgG antibody repertoires in patients with relapsing-remitting multiple sclerosis.. Immunology Letters.

[19] de Monte M, Nonnenmacher H, Brignon N, Ullmann M, Martin JP (2002). A multivariate statistical analysis to follow the course of disease after infection of cats with different strains of the feline immunodeficiency virus (FIV).. J Virol Methods.

[20] Dray S, Pettorelli N, Chessel D (2003). Multivariate Analysis of Incomplete Mapped Data.. Transactions in GIS.

[21] Everitt B, Casella G, Fienberg S, Olkin I (2005). An R and S-PLUS Companion to Multivariate Analysis;.

[22] Seber GAF (2004). Multivariate Observations..

[23] Gnanadesikan R (1997). Methods for Statistical Data Analysis of Multivariate Observations..

[24] Olson C (1976). On choosing a test statistic in multivariate analysis of variance.. Psychological Bulletin.

[25] Olson C (1974). Comparative Robustness of Six Tests in Multivariate Analysis of Variance.. Journal of the American Statistical Association.

[26] Krzanowski WJ (1988). Principles of Multivariate Analysis: a user's perspective..

[27] Fisher RA (1936). The Use of Multiple Measurements in Taxonomic Problems.. The Annals of Eugenics.

[28] Gollob KJ, Antonelli LR, Faria DR, Keesen TS, Dutra WO (2008). Immunoregulatory mechanisms and CD4-CD8- (double negative) T cell subpopulations in human cutaneous leishmaniasis: a balancing act between protection and pathology.. Int Immunopharmacol.

[29] Samaradasa W (2004). Generalized Inference in Repeated Measures: Exact Methods in MANOVA and Mixed Models..

[30] Ramsay J, Silverman BW (2005). Functional Data Analysis..

[31] McLachlan GJ (1992). Discriminant Analysis and Statistical Pattern Recognition..

[32] Dabo-Niang S, Ferraty F, Dabo-Niang S, Ferraty F (2008). Functional and Operatorial Statistics;.

